# Fasting and stimulated glucagon-like peptide-1 exhibit a compensatory adaptive response in diabetes and pre-diabetes states: A multi-ethnic comparative study

**DOI:** 10.3389/fendo.2022.961432

**Published:** 2022-09-09

**Authors:** Shiau Chin Chong, Norlela Sukor, Sarah Anne Robert, Kim Fong Ng, Nor Azmi Kamaruddin

**Affiliations:** ^1^ Department of Medicine, Universiti Kebangsaan Malaysia Medical Centre (UKMMC), Kuala Lumpur, Malaysia; ^2^ Department of Pharmacy, Universiti Kebangsaan Malaysia Medical Centre (UKMMC), Kuala Lumpur, Malaysia; ^3^ Department of Cardiology, Hospital Sultanah Aminah Johor Bahru, Johor, Malaysia

**Keywords:** glucagon-like peptide-1, pre-diabetes, type 2 diabetes mellitus, ethnicity, insulin resistance, incretin, glucose tolerance, secretion

## Abstract

**Background:**

Impaired secretion of glucagon-like peptide-1 (GLP-1) among Caucasians contributes to reduced incretin effect in type 2 diabetes mellitus (T2DM) patients. However, studies emanating from East Asia suggested preserved GLP-1 levels in pre-diabetes (pre-DM) and T2DM. We aimed to resolve these conflicting findings by investigating GLP-1 levels during oral glucose tolerance test (OGTT) among Malay, Chinese, and Indian ethnicities with normal glucose tolerance (NGT), pre-DM, and T2DM. The association between total GLP-1 levels, insulin resistance, and insulin sensitivity, and GLP-1 predictors were also analyzed.

**Methods:**

A total of 174 subjects were divided into NGT (n=58), pre-DM (n=54), and T2DM (n=62). Plasma total GLP-1 concentrations were measured at 0, 30, and 120 min during a 75-g OGTT. Homeostasis model assessment of insulin resistance (HOMA-IR), HOMA of insulin sensitivity (HOMA-IS), and triglyceride–glucose index (TyG) were calculated.

**Results:**

Total GLP-1 levels at fasting and 30 min were significantly higher in T2DM compared with pre-DM and NGT (27.18 ± 11.56 pmol/L vs. 21.99 ± 10.16 pmol/L vs. 16.24 ± 7.79 pmol/L, p=0.001; and 50.22 ± 18.03 pmol/L vs. 41.05 ± 17.68 pmol/L vs. 31.44 ± 22.59 pmol/L, p<0.001; respectively). Ethnicity was a significant determinant of AUC_GLP-1_, with the Indians exhibiting higher GLP-1 responses than Chinese and Malays. Indians were the most insulin resistant, whereas Chinese were the most insulin sensitive. The GLP-1 levels were positively correlated with HOMA-IR and TyG but negatively correlated with HOMA-IS. This relationship was evident among Indians who exhibited augmented GLP-1 responses proportionately to their high insulin-resistant states.

**Conclusion:**

This is the first study that showed GLP-1 responses are augmented as IR states increase. Fasting and post-OGTT GLP-1 levels are raised in T2DM and pre-DM compared to that in NGT. This raises a possibility of an adaptive compensatory response that has not been reported before. Among the three ethnic groups, the Indians has the highest IR and GLP-1 levels supporting the notion of an adaptive compensatory secretion of GLP-1.

## 1 Introduction

Type 2 diabetes mellitus (T2DM) is a major global public health problem. It is projected to affect 663 million people by 2030, with a significant proportion from Asia (1). Malaysia is unique because it is a multiracial country. The population of Malaysia consists of three major ethnic groups, namely, Malays, Chinese, and Indians. The prevalence of T2DM in Malaysia has risen from 13.4% in 2015 to 18.3% in 2019, with Indians having the highest prevalence (31.4%) followed by Malays (22.6%) and Chinese (15.1%) (2). It is also of a concern that the prevalence of pre-diabetes (pre-DM) is alarmingly high at 23.6% (2).

Glucagon-like peptide-1 (GLP-1) is a gastrointestinal hormone that is responsible for the incretin effect that potentiates insulin secretion following oral glucose loads (3). The impairment in GLP-1 secretion, especially post-prandial GLP-1 secretory response, has been shown to contribute to the development of T2DM (3). However, majority of the studies have focused on T2DM patients in the Western countries. Compared with Caucasians, T2DM in East Asians (with ethnic origin from China, Japan, South Korea, or Taiwan) are characterized primarily by impaired β-cell function, lower reserve capacity of insulin secretion, and higher insulin sensitivity (4). On the other hand, South Asians were found to have a greater degree of insulin resistance than Caucasians (5). Several East Asian studies have suggested that Korean and Japanese T2DM patients have comparable GLP-1 secretion as subjects with normal glucose tolerance (NGT) (6–9). In light of the marked inter-ethnic variation in the contribution of insulin secretion and insulin resistance, questions have been raised on whether reduced GLP-1 secretory response represents a universal characteristic of T2DM patients.

To the best of our knowledge, there has not been any studies looking at GLP-1 levels among the various ethnic groups in Malaysia. It is unclear whether disparities in GLP-1 secretion exists among the Malays, Chinese, and Indians in this country. In order to gain further insights into the role contributed by incretins in the pathophysiology of pre-DM and T2DM, our study aimed to analyze (1) the fasting GLP-1 levels and GLP-1 responses to oral glucose challenge test (OGTT) in various glucose tolerance states (NGT, pre-DM, and T2DM) and (2) the association between GLP-1 levels and insulin resistance (IR) and insulin sensitivity (IS) states among the three major ethnic groups.

## 2 Materials and methods

### 2.1 Study subjects

A total of 228 subjects aged 18 years old and above, who underwent routine health examination at various specialist clinics, were invited to participate in the study. For subjects with pre-existing T2DM who were on oral glucose lowering drugs (OGLDs) such as metformin and/or sulfonylureas and/or alpha-glucosidase inhibitors, they must be on a stable dose for at least 3 months prior to recruitment. At the screening visit, subjects’ ethnicities were determined based on their national identification cards. A 75-g OGTT was performed to categorize the patients into NGT, pre-DM, and T2DM groups. Blood samples were collected for hematological and biochemical assessment. Subjects with anemia (hemoglobin< 10 g/dl), renal impairment (serum creatinine>130 μmol/L), elevated liver enzymes, or subjects with chronic lung or heart diseases were excluded. Those with T2DM who possessed HbA1c>10% or fasting blood glucose>13 mmol/L or post-prandial blood glucose>18mmol/L (10), or if they were on medications that would influence the GLP-1 levels such as dipeptidyl peptidase-IV (DPP-IV) inhibitor or GLP-1 analogue or those who were on insulin were excluded from the study.

This study protocol was approved by the Research Ethics Committee of the National University of Malaysia (UKM PPI/111/8/JEP-2017-395) and the Medical Research & Ethics Committee, Ministry of Health Malaysia [NMRR-17-869-35075 (IIR)]. The study was registered at ClinicalTrials.gov (NCT03659461) and conformed to the Declaration of Helsinki. Written consent was obtained from all subjects.

### 2.2 Methods

#### 2.2.1 Study procedure

All subjects had their anthropometric and blood pressure measurements obtained in the morning. OGTT was performed following a 10-h fast. For subjects with T2DM, the last two doses of OGLDs were omitted prior to the conduct of the OGTT. At baseline, subjects had their blood drawn for blood glucose, HbA1c, insulin, GLP-1, total cholesterol, triglyceride, LDL, and HDL cholesterol levels. This was then followed by a 75-g glucose drink, which was consumed within 5 min. Subsequently, blood samples for measurement of glucose and GLP-1 levels were drawn at 30 and 120 min during the OGTT.

#### 2.2.2 Definition

The classification of glucose tolerance states were based on the OGTT diagnostic criteria outlined by the American Diabetes Association (11): NGT, fasting plasma glucose (FPG)<5.6 mmol/L and 2-h plasma glucose (2hPG)<7.8 mmol/L; pre-DM, FPG of 5.6–6.9 mmol/L or 2hPG of 7.8–11.0 mmol/L; T2DM, FPG ≥7.0 mmol/L or 2hPG ≥11.1 mmol/L.

In assessing insulin resistance and insulin sensitivity, triglyceride–glucose index (TyG), homeostasis model assessment of insulin resistance (HOMA-IR), and homeostasis model assessment of insulin sensitivity (HOMA-IS) were used.

TyG is a novel marker of insulin resistance and is calculated using the following formula (12):


TyG = ln [fasting gluclose (mg/dl) × triglycerides (mg/dl)/2]

HOMA-IR and HOMA-IS are robust surrogate methods to estimate insulin resistance and insulin sensitivity states in clinical setting. They are calculated using the following formulas (13, 14):


$$\text{HOMA-IR}=\frac{\text{fasting blood glucose (mmol/l) }\times \text{ fasting insulin (μU/ml)}}{22.5}$$

HOMA-IS = 1/HOMA-IR

#### 2.2.3 Biochemical measurement

HbA1c was measured using an automatic HbA1c analyzer (D10, Bio-Rad Laboratories, Hercules, CA, USA). The intra- and inter-assay coefficients of variation (CV) were 0.81 and 2.35% at HbA1c ≤ 6% and 0.48 and 1.65% at HbA1c ≥ 6.1%. The Roche Cobas 8000 Modular Analyzer (Basel, Switzerland) was used to measure plasma glucose (intra-assay CV<0.8% and inter-assay CV<1.4%) and total cholesterol, HDL cholesterol, and triglyceride levels (intra-assay CV<1.1% and inter-assay CV<2.1%). LDL cholesterol was calculated using Friedewald’s formula (15). The serum insulin was measured by an electrochemiluminescence immunoassay (Cobas E411, Roche Diagnostics, Mannheim, Germany). The detection limit was 0.2 μU/ml with inter-assay CV< 3.8%.

The total GLP-1 (7–36 and 9–36) was determined by enzyme-linked immunosorbent assay (EMD Millipore, Billerica, MA, USA) (16, 17). The plasma samples for GLP-1 determination were collected in an EDTA tube without any aprotinin, DPP-IV inhibitor, or anticoagulant. The tubes were kept on ice until centrifugation. After centrifugation at 3,000*g* for 15 min at 4°C, the samples were stored in aliquots at −80°C until further analysis in batches. The enzyme activity is measured spectrophotometrically by the increased absorbency at 450 nm, corrected from the absorbency at 590 nm, after acidification of formed products. Since the increase in absorbency is directly proportional to the amount of captured total GLP-1 in the unknown sample, the latter can be derived from the reference curve generated in the same assay with reference standards of known concentrations of GLP-1. The assay has a minimum detectable concentration of 1.5 pM/ml. The intra-assay CV was<2%, and the inter-assay CV was<12% (16, 17).

### 2.3 Statistical analyses and calculations

The sample size was estimated based on relevant effect sizes obtained from previous Asian study (6), with the calculated Cohen’s effect size of 0.52. By using a medium effect size of 0.4–0.5, a sample size with a statistical power of 0.90, and a p-value<0.05, the estimated number of subjects required was 16 for each ethnic groups that constituted NGT, pre-DM, and T2DM.

Data were analyzed using SPSS for windows version 23 (Chicago, IL, USA). All continuous variables were expressed as the mean ± SD or as median (25th percentile and 75th percentile) if the data were not normally distributed. One-way between-groups ANOVA with Bonferroni’s multiple comparison *post-hoc* test was used to compare mean differences in baseline characteristics among three glucose tolerance groups (NGT, pre-DM, and T2DM) or three ethnic groups (Malays, Chinese, and Indians), whereas independent Student’s t-test was used for comparison between two groups in normally distributed data. For non-parametric data, Kruskal–Wallis was used for comparison of mean differences among three groups, or Mann–Whitney test was employed to compare two groups. The chi-square test was used for categorical variables. Tests of differences in GLP-1 levels between glucose tolerance groups were adjusted for age, BMI, waist circumference, and waist-to-hip ratio. Spearman rank correlation analysis was used to explore correlations between GLP-1 levels with HOMA-IR, TyG, and HOMA-IS. Partial correlations were used to determine the association after adjusting for age, BMI, waist circumference, and waist-to-hip ratio. A p-value<0.05 was considered statistically significant.

Stepwise multiple linear regression analysis was performed to identify various clinical factors that affected GLP-1 levels. Multiple linear regression analysis was performed with area under the curve (AUC) GLP-1 as dependent variable. Independent variables were HOMA-IR, TyG, and HOMA-IS, and clinical factors included gender, ethnicity, smoking status, presence of family history of T2DM (first degree relatives), medical illnesses (hypertension and dyslipidemia), diabetic stages, age, weight, height, BMI, waist circumference, hip circumference, waist-to-hip ratio, HbA1c, systolic blood pressure, diastolic blood pressure, total cholesterol, HDL cholesterol, and LDL cholesterol. FPG and fasting insulin and fasting triglyceride could not be included in the analyses involving HOMA and TyG, respectively, due to multicollinearity. A step-by-step variable selection was carried out involving adding or removing potential independent variables in succession and examining for statistical significance after each iteration. A few models by using all or different combinations of independent variables were constructed. The best final model that accounted for the most variance in the dependent variable (adjusted r^2^) was chosen.

The AUCs were estimated according to the trapezoidal rule. The incremental GLP-1 (ΔGLP-1) levels were calculated by subtracting the fasting GLP-1 level from the GLP-1 levels at 30 and 120 min.

## 3 Results

**Figure 1:**
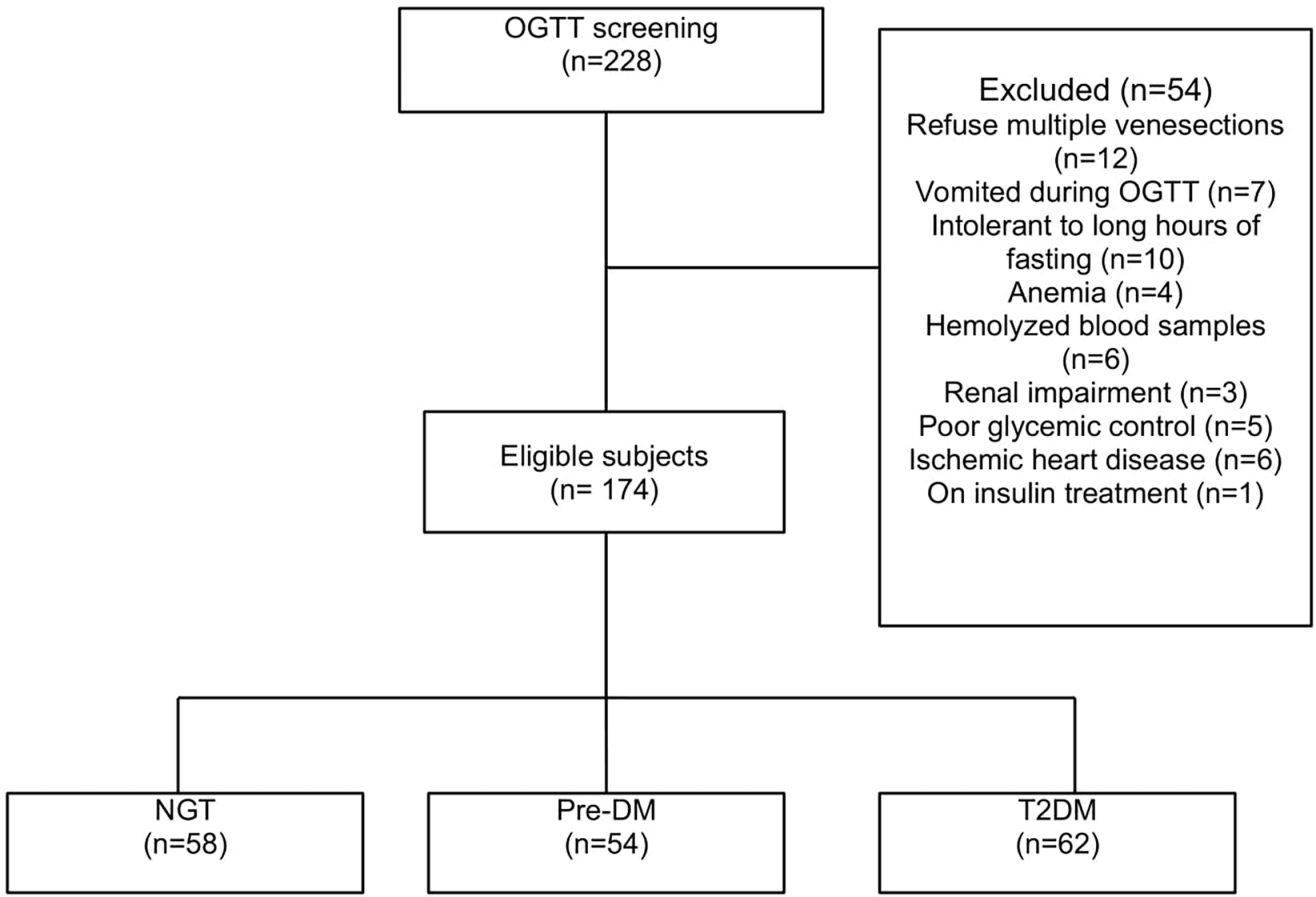
Patient disposition.

A total of 174 subjects who fulfilled the study criteria were included in this study. Out of the 174 subjects, 62 were in the T2DM group, 54 in the pre-DM group, and 58 belonged to the NGT group (**Figure 1**).

For the T2DM group, 33 subjects were known to have diabetes, whereas 29 were newly diagnosed. Those with history of diabetes had a mean disease duration of 6.2 ± 5.8 years (mean ± SD). Thirty subjects were treated with OGLDs: metformin [n = 12], sulfonylurea [n = 1], metformin and sulfonylureas [n = 15], metformin, sulfonylurea and acarbose [n = 1], and metformin and acarbose [n = 1], while the remaining three were drug naive.

### 3.1 Baseline characteristics

**Table 1** shows the baseline clinical characteristics of subjects across all glucose tolerance states. Compared with NGT and subjects with pre-DM, T2DM subjects were older and had higher BMI, waist circumference, hip circumference, waist-to-hip ratio, systolic and diastolic blood pressure, HbA1c, fasting glucose, fasting insulin, and triglyceride and lower HDL cholesterol levels. The majority of T2DM patients had hypertension and dyslipidemia. There were no significant differences in all parameters of clinical characteristics among Malays, Chinese, and Indians except that Chinese were older compared to Indians and Malays (**Table 2**). Indians had significantly higher fasting insulin (17.43 ± 7.52 μU/ml) than Chinese (14.43 ± 11.99 μU/ml) and Malays (14.21 ± 11.1 μU/ml).

**Table 1:** Clinical characteristics of subjects with NGT, pre-DM, and T2DM.

	NGT (n = 58)	Pre-DM (n = 54)	T2DM (n = 62)	p-value
Male/Female	21/37	26/28	32/30	0.211
Age (years)	32 (27, 48)*ƚ	52 (40, 60)ƚ	57 (47, 63)	<0.001
Family history of diabetes (first-degree relatives)	26 (44.8)	28 (51.9)	39 (62.9)	0.134
Medical history, n (%) Smoker Hypertension Dyslipidemia	3 (5.2) 7 (12.1) 5 (8.6)	9 (16.7) 14 (25.9) 18 (33.3)	11 (17.7) 36 (58.1) 23 (37.1)	0.085<0.001 0.001
Weight (kg)	63.7 (53.5, 79)	69.7 (61.8, 76.9)	68.9 (63.8, 83.4)	0.28
Height (cm)	161.1 (155.9, 168.2)	160.4 (154.3, 170.3)	160.8 (154.1, 167.5)	0.77
Body mass index (kg/m^2^)	24 (21.7, 28)*ƚ	26.2 (24.4, 29.8)	26.7 (24.6, 31.8)	0.001
Waist circumference (cm)	87.3 (80.5, 94.3)*ƚ	93.5 (87.8, 100.25)	95 (90, 108.75)	<0.001
Hip circumference (cm)	101 (92.9, 107.3)*ƚ	104.8 (99.8, 110)	105 (98, 111.3)	0.012
Waist-to-hip ratio	0.85 (0.79, 0.92)*ƚ	0.88 (0.8, 0.94)	0.93 (0.87, 0.98)	0.001
Systolic blood pressure (mmHg)	119 ± 14*ƚ	131 ± 17	132 ± 17	<0.001
Diastolic blood pressure (mmHg)	74 ± 9*ƚ	78 ± 11(70,85)	79 ± 13	0.012
**Laboratory assay**				
HbA1c (%)	5.3 (5.1, 5.6)*ƚ	6 (5.6, 6.3)ƚ	7.1 (6.5,7.9)	<0.001
Fasting insulin (µU/ml)	10.8 ± 5.77*ƚ	16.33 ± 8.19	18.46 ± 13.98	<0.001
Triglyceride (mmol/L)	0.85 (0.68, 1.33)*ƚ	1.1 (0.9, 1.83)ƚ	1.55 (1.2, 2.03)	<0.001
Total cholesterol (mmol/L)	5.18 (4.2, 5.6)	5.05 (4.58, 5.5)	4.8 (4.08, 5.63)	0.553
LDL-C(mmol/L)	2.96 ± 0.73	3.08 ± 0.92	2.81 ± 1.27	0.27
HDL-C (mmol/L)	1.5 (1.3, 1.8)*ƚ	1.25 (1,1.53)	1.2 (1, 1.3)	<0.001
Fasting glucose (mmol/L)	4.95 ± 0.44*ƚ	5.71 ± 0.71ƚ	7.56 ± 2.23	<0.001

Data are expressed as mean ± standard deviation or median (25th percentile and 75th percentile).

NGT, normal glucose tolerance; pre-DM, pre-diabetes; T2DM, type 2 diabetes mellitus; LDL-C, low-density lipoprotein cholesterol; HDL-C, high-density lipoprotein cholesterol; HOMA-IR, homeostasis model assessment of insulin resistance; HOMA-IS, homeostasis model assessment of insulin sensitivity; TyG, triglyceride–glucose index.

*p<0.05 versus pre-DM; ƚ p<0.05 versus T2DM.

**Table 2:** Clinical characteristics of Malay, Chinese, and Indian subjects with NGT, pre-DM, and T2DM.

	Malays (n = 62)				Chinese (n = 60)				Indians (n = 52)				Overall
	NGT (n = 19)	pre-DM (n = 21)	T2DM (n = 22)	p-value	NGT (n = 20)	pre-DM (n = 17)	T2DM (n = 23)	p-value	NGT (n = 19)	pre-DM (n = 16)	T2DM (n = 17)	p-value	p-value^a^
Male/female	6/13	9/12	11/11	0.489	10/10	9/8	13/10	0.912	5/14	8/8	8/9	0.288	0.309
Age (years)	27 (25, 32)*ƚ	43 (34.5, 54)	54 (45, 61)	<0.001	35 (27, 61)*ƚ	59 (45, 63)	58 (47, 65)	0.045	37 (30, 48)*ƚ	53 (38, 59)	58 (50, 63)	<0.001	0.003
Family history of diabetes (first-degree relatives)	8 (42.1)	10 (47.6)	11 (50)	0.876	7 (35)	11 (64.7)	15 (65.2)	0.088	11 (57.9)	7 (43.8)	13 (76.5)	0.157	0.368
Medical history, n (%) Smoker Hypertension Dyslipidemia	1 (5.3) 1 (5.3) 1 (5.3)	5 (23.8) 6 (28.6) 4 (19)	3 (13.6) 11 (50) 7 (31.8)	0.323 0.007 0.117	2 (10) 3 (15) 3 (15)	1 (5.9) 7 (41.2) 8 (47.1)	6 (26.1) 17 (73.9) 11 (47.8)	0.222 0.001 0.048	0 (0) 3 (15.8) 1 (5.3)	3 (18.8) 1 (6.3) 6 (37.5)	2 (11.8) 8 (47.1) 5 (29.4)	0.144 0.021 0.045	0.655 0.35 0.077
Weight (kg)	59ƚ (53.5,79)	68.3 (60.1, 75.5)	75.5 (63, 92.7)	0.033	64.2 (51.7, 73.6)	66 (58.1, 75.9)	67.2 (63.8, 77.7)	0.213	73.9 (57.7, 82.8)	70.8 (68.9, 77.8)	67.7 (61.2, 83.2)	0.826	0.245
Height (cm)	160 ± 7.59	159.8 ± 9.2	158.2 ± 8.6	0.750	163.4 ± 10.1	162 ± 9.7	162.8 ± 7.5	0.931	163.2 ± 7.6	163.4 ± 8.3	162.4 ± 10.5	0.943	0.34
Body mass index (kg/m^2^)	23.86 (22.03, 27.12)ƚ	27.18 (24.13, 29.86)	29.15 (24.76, 36.74)	0.008	23.38 (19.84, 25.24)*ƚ	25.95 (22.87, 27.91)	25.76 (24.4, 28.58)	0.007	26.36 (22.26, 29.72)	26.59 (24.58, 31.05)	26.31 (24.73, 31.56)	0.853	0.21
Waist circumference (cm)	84 (79, 96)ƚ	93 (86.5, 101)	103 (90.3, 110.5)	0.009	93.5 (87.8, 96)*ƚ	93 (86.5, 101)	92 (90, 99)	0.042	89 (83, 95)*ƚ	97 (89.1, 100.8)	96 (89.5, 109)	0.014	0.428
Hip circumference (cm)	104 (98, 108)	103 (98, 109.8)	107.5 (99.5, 115)	0.212	95.4 (86.3, 100.5)*ƚ	104 (98.8, 109.5)	100 (97,109)	0.005	106 (95, 114)	110 (103.6, 118.3)	105 (101, 112.3)	0.262	0.38
Waist-to-hip ratio	0.82 (0.78, 0.89)ƚ	0.89 (0.78, 0.97)	0.93 (0.85, 0.99)	0.022	0.89 (0.82, 0.98)	0.88 (0.8, 0.95)	0.93 (0.87, 0.97)	0.464	0.84 (0.8, 0.88)ƚ	0.88 (0.78, 0.91)	0.91 (0.87, 0.97)	0.028	0.22
Systolic blood pressure (mmHg)	118 ± 9ƚ	129 ± 20	133 ± 18	0.012	122 ± 16ƚ	132 ± 18	137 ± 14	0.009	118 ± 16*	134 ± 13	124 ± 17	0.017	0.22
Diastolic blood pressure (mmHg)	73 ± 7	75 ± 12	82 ± 14	0.064	73 ± 9*ƚ	79 ± 9	81 ± 10	0.02	74 ± 11	81 ± 8**ƚ**	73 ± 12	0.085	0.488
Heart rate	80 ± 14	75 ± 11	84 ± 16	0.141	76 ± 10	78 ± 15	76 ± 15	0.904	72 ± 11	72 ± 12	73 ± 11	0.883	0.05
**Laboratory assay**													
HbA1c (%)	5.2 (5, 5.5)*ƚ	5.8ƚ (5.2,6.3)	7 (6.4,7.7)	<0.001	5.3 (5.1, 5.7)*ƚ	6 (6, 6.6)ƚ	7 (6.4, 7.7)	<0.001	5.4 (5.1, 5.7)*ƚ	6.1 (5.9, 6.4)ƚ	7.7 (7, 8.9)	<0.001	0.368
Fasting insulin (µU/ml)	9.77 ± 4.54	16.02 ± 10.10	16.31 ± 14.73	0.111	8.44 ± 3.82*ƚ	13.27 ± 7.84	20.49 ± 15.99	0.03	14.32 ± 7.01*	20 ± 3.18	18.5 ± 9.8	0.062	0.002
Triglyceride (mmol/L)	0.8 (0.6, 1.3)*ƚ	1.3 (1, 2.1)	1.7 (1.2, 2.2)	0.001	0.9 (0.6, 1.4)ƚ	1.1 (0.9, 1.8)ƚ	1.5 (1.2, 2.1)	0.008	0.9 (0.8, 1.4)ƚ	1 (0.9, 1.6)ƚ	1.3 (1.2, 1.8)	0.028	0.825
Total cholesterol (mmol/L)	4.9 (4,5.6)	5.2 (4.7, 5.5)	5.4 (4.2,5.9)	0.825	5.1 (4.2, 5.4)	4.9 (4.3, 5.5)	4.8 (3.8, 5.5)	0.835	5.3 (4.6, 5.6)	5 (4.7, 5.5)	4.6 (4.2, 5.1)	0.141	0.279
LDL-C (mmol/L)	2.9 ± 0.7	3.2 ± 0.9	3.1 ± 1.2	0.611	2.8 ± 0.8	2.8 ± 1.1	2.6 ± 1	0.646	3.2 ± 0.6	3.3 ± 0.7	2.8 ± 1	0.15	0.06
HDL-C (mmol/L)	1.5 (1.4, 1.6)*ƚ	1.1 (1,1.5)	1.3 (1.1, 1.4)	0.01	1.6 (1.1, 2)ƚ	1.4 (1.1, 1.8)	1.2 (1, 1.3)	0.04	1.5 (1.3, 1.7)ƚ	1.2 (1, 1.4)	1.1 (1, 1.3)	0.044	0.606

Data are expressed as mean ± standard deviation or median (25th percentile and 75th percentile).

NGT, normal glucose tolerance; pre-DM, pre-diabetes; T2DM, type 2 diabetes mellitus; LDL-C, low-density lipoprotein cholesterol; HDL-C, high-density lipoprotein cholesterol.

a Malay versus Chinese versus Indian.

*p<0.05 versus pre-DM; ƚ p<0.05 versus T2DM.

### 3.2 Glucose levels during OGTT

**Figure 2:**
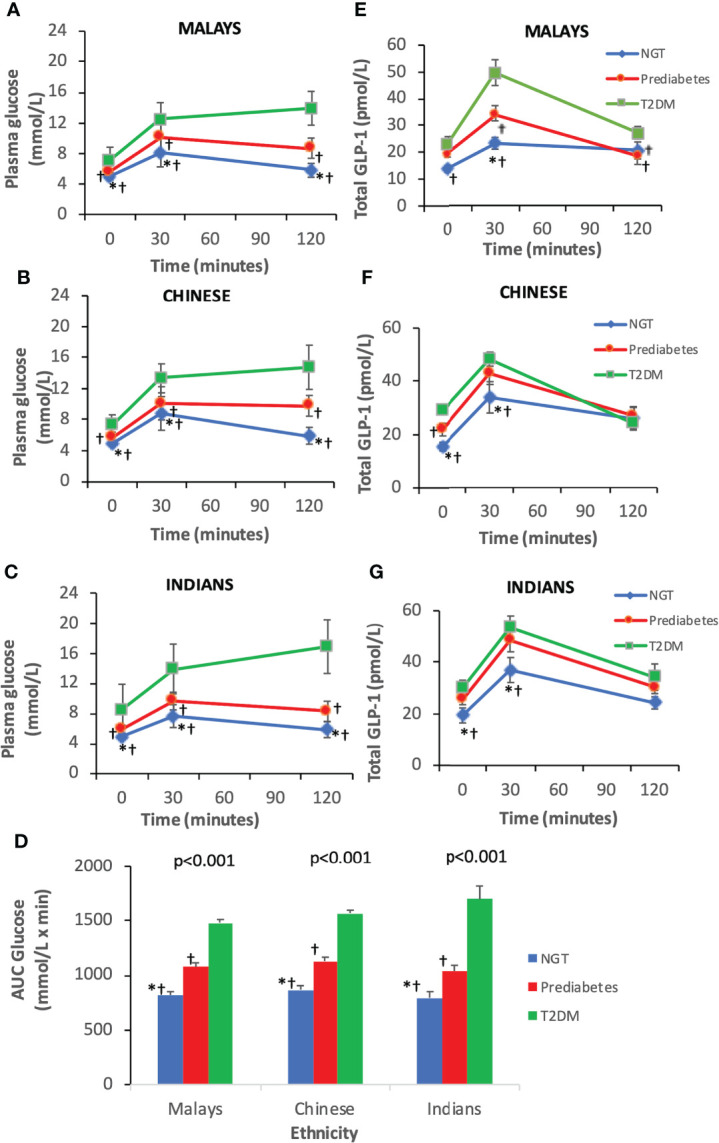
Time courses for plasma concentrations of glucose and glucagon-like peptide-1 (GLP-1) during an oral glucose tolerance test by ethnicity. Data are expressed as mean ± SD for **(A–C)** glucose levels, **(D)** AUC_Glucose_, and **(E–G)** total GLP-1 levels. *p < 0.05 versus pre-diabetes, ^ƚ^p < 0.05 versus T2DM. AUC, area under the curve; NGT, normal glucose tolerance; T2DM, type 2 diabetes mellitus.

**Figure 3:**
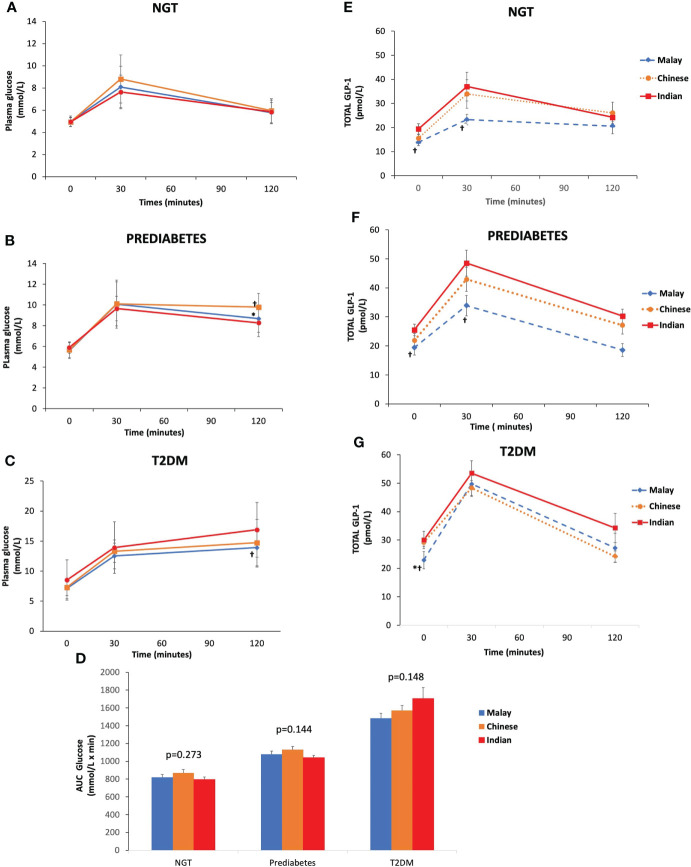
Time courses for plasma concentrations of glucose and glucagon-like peptide-1 (GLP-1) during an OGTT by glucose tolerance states. Data are expressed as mean ± SD for **(A–C)** glucose levels and **(D)** AUC_Glucose_ and **(E–G)** total GLP-1 levels. *p < 0.05 versus Chinese, ^ƚ^p < 0.05 versus Indians. AUC, area under the curve; NGT, normal glucose tolerance; T2DM, type 2 diabetes mellitus.

As expected, T2DM subjects had higher plasma glucose levels at fasting, 30 and 120 min than pre-DM and NGT across three ethnic groups (**Figures 2A–C**). Similarly, the AUC_Glucose_ was greatest in T2DM, followed by pre-DM and NGT (**Figure 2D**). The plasma glucose levels at fasting and 30 min were similar across ethnicities among T2DM, pre-DM, and NGT groups (**Figures 3A–C**). The AUC_Glucose_ showed a similar trend across all glucose tolerance states among the ethnic groups (**Figure 3D**).

### 3.3 Total GLP-1 levels during OGTT

The T2DM group had significantly higher fasting total GLP-1 level than pre-DM and NGT groups (**Table 3**). Peak GLP-1 secretion occurred at 30 min during OGTT. The T2DM group showed a higher GLP-1 response at 30 min compared with pre-DM and NGT. The GLP-1 levels gradually decreased with time. This contributed to a significantly higher ΔGLP-1_30 min_ in the T2DM group than in the pre-DM and NGT groups. No significant differences were observed in GLP-1 levels at 120 min among NGT, pre-DM, and T2DM groups. However, NGT showed the greatest ΔGLP-1_120 min_ followed by pre-DM and T2DM groups. The AUC_GLP-1_ was 44% higher in the T2DM group relative to the NGT group (p<0.001) and 18% higher in the T2DM subjects than in pre-DM (p=0.004).

**Table 3:** Comparisons of total GLP-1 levels, insulin resistance, and insulin sensitivity indices in NGT, pre-DM, and T2DM.

Variables	NGT (n = 58)	Pre-DM (n = 54)	T2DM (n = 62)	p-value
**GLP-1**				
FGLP-1 (pmol/L)	16.24 ± 7.79*ƚ	21.99 ± 10.16 ƚ	27.18 ± 11.56	0.001
GLP-1_30 min_ (pmol/L)	31.44 ± 22.59*ƚ	41.05 ± 17.68 ƚ	50.22 ± 18.03	<0.001
GLP-1_120 min_ (pmol/L)	23.73 ± 12.51	24.97 ± 11.58	27.91 ± 14.03	0.512
ΔGLP-1_30 min_ (pmol/L)	15.2 ± 19.36*ƚ	18.76 ± 14.23	22.94 ± 16.84	0.018
ΔGLP1_120 min_ (pmol/L)	7.66 ± 13.12*ƚ	2.18 ± 11.04	0.31 ± 13.98	0.041
AUC_GLP-1_ (pmol//L.min)	3,265.94 ± 1953*ƚ	3,994.45 ± 1541 ƚ	4,698.22 ± 1542	<0.001
**Insulin resistance/sensitivity**				
HOMA-IR	2.4 ± 1.35*ƚ	4.23 ± 2.24 ƚ	6.2 ± 4.68	<0.001
TyG	8.22 ± 0.52*ƚ	8.64 ± 0.57 ƚ	9.14 ± 0.61	<0.001
HOMA-IS	0.55 ± 0.33*ƚ	0.35 ± 0.29 ƚ	0.28 ± 0.26	<0.001

Data are expressed as mean ± standard deviation.

GLP-1, glucagon-like peptide-1; FGLP-1, fasting GLP-1; ΔGLP-1, incremental GLP-1; HOMA-IR, homeostasis model assessment of insulin resistance; HOMA-IS, homeostasis model assessment of insulin sensitivity; TyG, triglyceride–glucose index; AUC, area under the curve; NGT, normal glucose tolerance; pre-DM, pre-diabetes; T2DM, type 2 diabetes mellitus.

*p<0.05 versus pre-DM; ƚ p< 0.05 versus T2DM.

Similar trends were observed in all three ethnic groups where T2DM exhibited higher fasting and 30-min GLP-1 levels than pre-DM and NGT, respectively (**Figures 2E–G**). With regards to the inter-ethnic differences, in all glucose tolerance states (T2DM, pre-DM, and NGT), the fasting total GLP-1 levels were significantly higher in Indians compared to Malays (30 ± 12.57 pmol/L vs. 22.95 ± 13.69 pmol/L, p<0.05; 25.48 ± 7.79 pmol/L vs. 19.41 ± 11.58 pmol/L, p<0.05; 19.39 ± 9.4 pmol/L vs. 13.82 ± 5.61 pmol/L, p<0.05), respectively. The fasting total GLP-1 values in Chinese were in between that of the Indians and Malays with no statistical difference noted (**Figures 3E–G**).

### 3.4 Insulin resistance (HOMA-IR, TyG) and insulin sensitivity (HOMA-IS)

**Figure 4:**
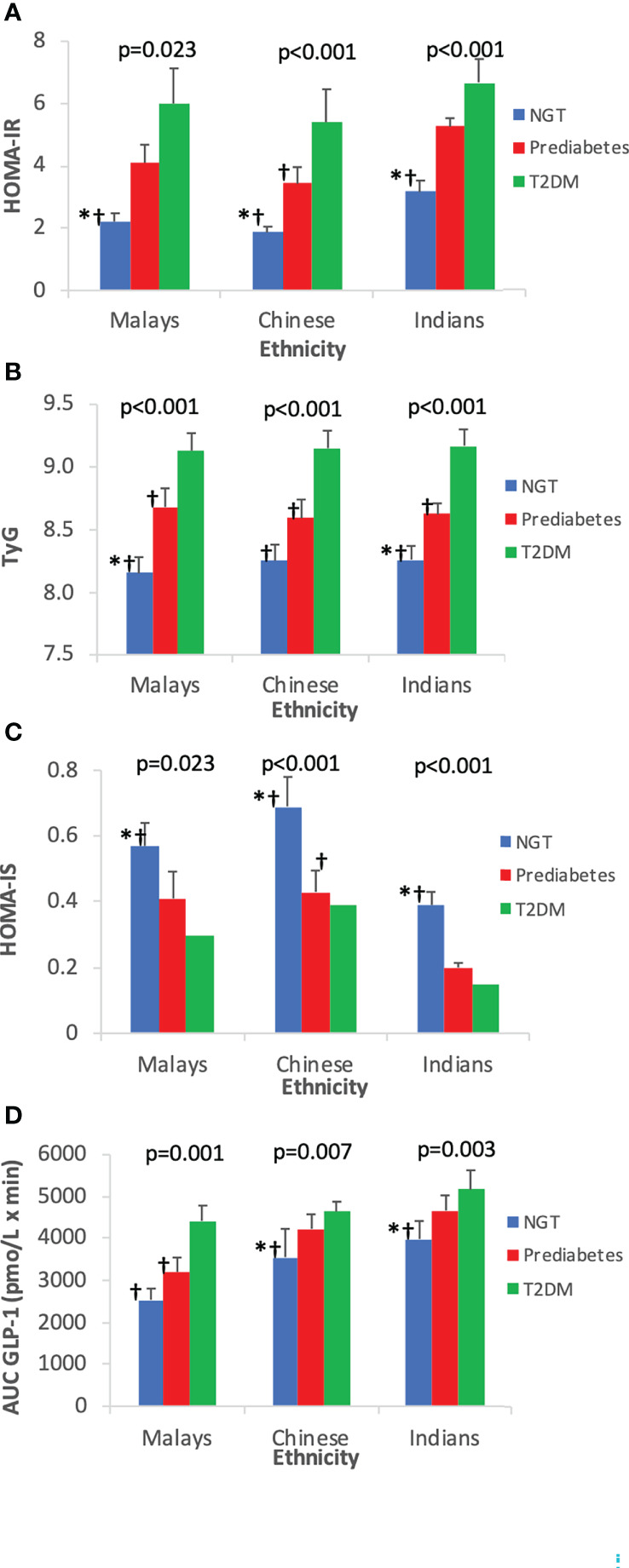
Insulin resistance and insulin sensitivity indices by ethnicity. Data are expressed as mean ± SD for **(A)** HOMA-IR, **(B)** TyG, **(C)** HOMA-IS, and **(D)** AUC_GLP-1_. *p < 0.05 versus pre-diabetes, ^ƚ^p < 0.05 versus T2DM. NGT, normal glucose tolerance; T2DM, type 2 diabetes mellitus; HOMA-IR, homeostasis model assessment of insulin resistance; HOMA-IS, homeostasis model assessment of insulin sensitivity; TyG, triglyceride–glucose index.

The IR at fasting state as expressed by HOMA-IR and TyG showed that T2DM subjects were the most insulin resistant compared with pre-DM and NGT subjects (**Table 3**). With regards to HOMA-IS, NGT had the highest level followed by pre-DM and T2DM. Indians had the highest insulin-resistant state and the lowest HOMA-IS than the Malays and Chinese, respectively (**Figures 4A–C**).

**Figure 4D** shows the increasing trend of AUC_GLP-1_ across increasing glucose tolerance state. Among the three ethnic groups, Indians who had the highest IR exhibited the highest GLP-1 responses compared with Chinese and Malays.

### 3.5 Correlation of GLP-1 with IR and IS

There was a positive correlation between fasting GLP-1 level and fasting insulin level (r=0.547, p<0.001). Total GLP-1 levels at each time point, ΔGLP-1_30 min_, ΔGLP-1_120 min_, and AUC_GLP-1_ were positively correlated with HOMA-IR and TyG but negatively correlated with HOMA-IS (**Table 4**).

**Table 4:** Correlation analysis of GLP-1 with HOMA-IR, HOMA-IS, TyG, and AUC glucose.

Variables	HOMA-IR		HOMA-IS		TyG		AUC_Glucose_	
	r	p-value	r	p-value	r	p-value	r	p-value
FGLP-1	0.576	<0.001	−0.577	<0.001	0.47	<0.001	0.427	<0.001
GLP-1_30 min_	0.345	<0.001	−0.346	<0.001	0.457	<0.001	0.521	<0.001
GLP-1_120 min_	0.178	0.026	−0.178	0.027	0.175	0.029	0.171	0.032
ΔGLP-1_30 min_	0.018	0.809	−0.019	0.802	0.237	0.002	0.344	<0.001
ΔGLP-1_120 min_	0.374	<0.001	−0.376	<0.001	0.283	<0.001	0.281	<0.001
AUC_GLP-1_	0.325	<0.001	−0.327	<0.001	0.400	<0.001	0.486	<0.001

r, Spearman correlation coefficient; GLP-1, glucagon-like peptide-1; FGLP-1, fasting GLP-1; ΔGLP-1, incremental GLP-1; HOMA-IR, homeostasis model assessment of insulin resistance; HOMA-IS, homeostasis model assessment of insulin sensitivity; TyG, triglyceride–glucose index; AUC, area under the curve.

### 3.6 Multivariate linear regression analysis

The correlation among AUC_GLP-1_, IR, and IS were confirmed by multiple linear regression analysis. The correlation between AUC_GLP-1_ and HOMA-IR, and TyG and HOMA-IS remained significant. There was a positive correlation between increasing age and AUC_GLP-1_. Ethnicity was a significant determinant of AUC_GLP-1_, with the Indians exhibiting higher GLP-1 secretion than the Malays. AUC_GLP-1_ was positively associated with increased systolic blood pressure in the model that included HOMA-IR, but the relationship was insignificant in the TyG and HOMA-IS models (**Table 5**).

**Table 5:** Multiple linear regression analysis of AUC_GLP-1,_ with HOMA-IR, TyG, HOMA-IS, and other independent variables.

Parameters	B	p-value
Model with HOMA-IR as independent variable (adjusted r^2 =^ 0.273)		
HOMA-IR	65.68	0.004
Age	51.21	<0.001
Gender Male Female	– –781.62	– 0.063
Ethnicity Malays^a^ Chinese Indians	– −18.75 720.19	– 0.955 0.008
Waist circumference	110.42	0.115
Systolic blood pressure	16.61	0.031
Total Cholesterol	542.57	0.051
Model with TyG as independent variable (adjusted r^2 =^ 0.274)		
TyG	631.36	0.001
Age	47.19	<0.001
Ethnicity Malays^a^ Chinese Indians	– −16.52 746.68	– 0.745 0.005
Systolic blood pressure	13.44	0.052
Model with HOMA-IS as independent variable (adjusted r^2^ = 0.231)		
HOMA-IS	−1,160.01	0.004
Age	53.95	<0.001
Ethnicity Malays^a^ Chinese Indians	– −67.25 651.03	– 0.536 0.001
Systolic blood pressure	12.89	0.063

The results are presented as unstandardized coefficient, B- and p-value using stepwise multiple linear regression.

a Malay was assigned as the reference group from which Chinese and Indians were compared.

## 4 Discussion

This study was conducted to determine the fasting GLP-1 levels and the GLP-1 responses following OGTT with the 75-g glucose in NGT, pre-DM, and T2DM. In addition, indices of IR (HOMA-IR and TyG) and IS (HOMA-IS) were also determined in the three cohorts of glucose tolerance states. Subsequently, all of the above parameters involving GLP-1, IR, and IS were further analyzed within the three main ethnic groups consisting of Malays, Chinese, and Indians, primarily to discern any potential differences between the ethnicities.

The glucose tolerance status of the three groups were further established by assessing the HOMA-IR, TyG, and HOMA-IS. In this respect, T2DM had the highest IR (HOMA-IR, TyG) and lowest IS (HOMA-IS). NGT had the lowest IR and highest IS, while pre-DM had their IR and IS that fell in between the two cohorts.

### 4.1 Fasting GLP-1 levels and GLP-1 responses to OGTT

A novel finding of our study is that the total GLP-1 levels increased as the IR state increased (from NGT to pre-DM and T2DM). The fasting total GLP-1 levels in our cohorts were the highest in T2DM followed by pre-DM and NGT, demonstrating an increment of the fasting GLP-1 levels as the glucose tolerance states progressed. Similarly, the post-challenge GLP-1 levels at 30 min were the highest in T2DM compared to pre-DM and NGT. This is further supported by both the ΔGLP-1_30_ and AUC_GLP-1_ being the highest in the T2DM compared to the other groups.

Our finding is in contrast to other studies that reported insulin resistance impairs GLP-1 secretion (18–21). Studies conducted in the Western population showed that GLP-1 levels tend to fall as glucose tolerance state worsens (22–25). The more recent Denmark ADDITION-PRO study, which was one of the largest longitudinal risk-stratified cohort study, demonstrated that total GLP-1 responses, especially GLP-1 responses at 120 min during OGTT, were reduced by 16%–21% in pre-DM and T2DM compared to subjects with NGT (24). The first prospective cohort study, which investigated the association between total GLP-1 response to an OGTT at baseline and changes in fasting glucose levels 7 years later in individuals who were non-diabetics at baseline, found that the GLP-1 secretion reduced in individual whose fasting glucose increased over time (25). Therefore, reduced GLP-1 secretion precedes glucose deterioration, and this deficiency in GLP-1 secretion partly contribute to the diminished incretin effect typically observed among Caucasians with T2DM.

It is well recognized that Asians develop T2DM at a younger age with lower body mass index and more visceral fat as compared with the Western populations (26). Hence, ethnic differences might contribute to the varying GLP-1 secretory responses. In contrast to Western studies, several studies in East Asia showed the GLP-1 levels were not reduced in patients with pre-DM or T2DM. Japanese T2DM patients exhibited similar GLP-1 responses as non-diabetics after oral glucose and meal challenge tests (6, 7, 27). Likewise, studies conducted in Korea also showed similar findings of sustained GLP-1 levels following the development of T2DM (8, 9) Therefore, the authors concluded that GLP-1 secretion deficiency does not account for the reduced insulin secretory capacity in Japanese and Koreans. The reasons for the inconsistent results observed are still unclear; however, several factors have been postulated, which include the different diagnostic criteria used to diagnose T2DM and pre-DM, smaller sample size, timing of GLP-1 measurements, glucose challenge used (OGTT or mixed meals), GLP-1 detection method, racial and genetic differences (18, 19, 26, 28, 29).

A novel finding of our study is that the total GLP-1 levels were positively correlated with the insulin resistance. In order to maintain glucose homeostasis with declining insulin sensitivity, a proportionate increase in insulin release occurs as a compensatory mechanism. Our study showed in pre-DM and T2DM groups their early-phase (0–30 min) GLP-1 secretion were markedly increased, and this represents a compensatory mechanism, as reflected by their significantly higher fasting insulin levels but comparable glucose levels when compared with NGT.

A meta-analysis by Calanna et al. (28) investigating the GLP-1 levels in diabetic versus non-diabetic controls encompassing 22 trials using 29 different stimulation tests revealed that patients with T2DM exhibited similar responses of total GLP-1 to the stimulation tests compared with non-diabetic controls. The authors suggested that compensatory L-cell secretion of GLP-1 may occur in the early stages of T2DM, followed by L-cell exhaustion as the disease progresses. According to Theodorakis et al., the early rise in GLP-1 level following OGTT was related to upregulation of L-cells in the duodenum in new onset T2DM (30). These findings are in line with our study since as many as 47% of our T2DM subjects were newly diagnosed. In this study, the pre-DM had significantly higher GLP-1 secretion than NGT. An earlier study also revealed that fasting GLP-1 level was significantly increased among pre-DM (31). It is therefore worth noting that increased GLP-1 level reflects compensatory adaptive response, and this occurs even before T2DM manifests in an attempt to overcome the progressive loss in β-cell response. The incretin effect is reduced significantly in patients with T2DM from 70% to 20%–35% compared to individuals with NGT (3, 32). Nevertheless, it is noteworthy that the defective incretin effects occurred even with preserved or increased GLP-1 levels as being demonstrated by our finding and other studies (6–9, 27).

There are several possible mechanisms leading to elevated GLP-1 levels seen in T2DM. These include accelerated gastric emptying rate (22, 23); the use of metformin, which has been reported to increase plasma GLP-1 concentrations through stimulation of GLP-1 secretion and reduction in soluble DPPIV-activity (33, 34); the type of stimulus use for example in this study, OGTT was utilized. Previous studies have shown that the type of stimulus used can potentially affect GLP-1 secretion. GLP-1 responses increased following liquid mixed meal tests and OGTT but decreased following solid mixed meal tests (7, 28, 35). We also hypothesize that the increased GLP-1 levels in our T2DM subjects might indicate a state of GLP-1 resistance, since hyperglycemia has been proposed to downregulate GLP-1 receptor expression on β cells and cause GLP-1 resistance (5, 28, 32, 36).

Consistent with previous studies, our study found that the GLP-1 level increased and reached the peak 30 min after glucose loads (6, 20). It is nevertheless noteworthy that this compensatory GLP-1 secretion gradually diminished at late phase in T2DM group, as reflected by the decrease in GLP-1 concentration at 120 min reaching the fasting GLP-1 level. As a result, the ability of β cells to secrete sufficient insulin to adequately reduce the glucose load at the prevailing peripheral insulin resistance state appears to progressively diminish. Subsequently, we observed an increase in plasma glucose concentration at 120 min during OGTT. Our finding of lower 120-min GLP-1 response is in agreement with previous studies, which also observed reduced GLP-1 responses after 120 min in pre-DM and T2DM (22, 24). In addition, increasing HbA1c level is negatively associated with GLP-1 response (28). In line with this, the GLP-1 secretion profile could be altered as diabetes progresses. Despite our finding suggested compensatory GLP-1 secretion exists in pre-DM and early stage of T2DM, we hypothesize that this compensatory response may eventually fail due to “exhaustion” of L cells with disease progression and deterioration of glycemic control.

### 4.2 Insulin resistance and insulin sensitivity

T2DM is characterized by IR and impaired insulin secretion due to β-cell failure (26). IR was measured using HOMA-IR and TyG. TyG was used as one of the surrogate markers of insulin resistance in our study because it has been proposed that it can be reliably used in Asian T2DM patients who were on oral hypoglycaemic treatment (37). As expected, both markers provide similar information, where insulin resistance increased significantly from NGT to pre-DM and from pre-DM to T2DM.

### 4.3 Ethnicity differences

In general, Indians had the highest GLP-1 responses, followed by Chinese and Malays. These ethnic differences in circulating GLP-1 levels were particularly more pronounced in NGT and pre-DM groups. Indians were the most insulin resistant, whereas Chinese appeared to be more insulin sensitive than Malays. Contrary to other studies, our findings showed significant positive correlation between GLP-1 responses and insulin resistance. The GLP-1 response reflected the prevailing IR state. This relationship was evident among Indians who exhibited augmented GLP-1 responses proportionately to their high insulin-resistant states. We hypothesize that compensatory increased GLP-1 responses could possibly explain these findings in our study population especially in Indians to overcome their on-going insulin resistance.

However, comparison among three ethnicities showed varying degrees of IR states, either measured by HOMA-IR or TyG. HOMA-IR was significantly higher in Indians than in Malays and Chinese regardless of glucose tolerance states. However, when measured with TyG, the three ethnic groups had comparable degree of IR states. This might be attributed to similar triglyceride profiles among the different ethnic groups (38). As opposed to HOMA-IR, Chinese were more insulin sensitive (as indicated by HOMA-IS) than Malays and Indians. There is a profound variation in the degree of IR among East Asians, South Asians, and Caucasians. It has been proposed that East Asians (Japanese, Chinese, or Koreans) have lower IR compared with Caucasians (39). On the other hand, South Asians are more insulin resistant than Caucasians (5). Indeed, in accordance with the findings from these aforementioned studies, our study yielded similar results where Indians were the most insulin resistant, Chinese were the most insulin sensitive, whereas Malays were intermediate. The findings of our study are also in agreement with another Asian-based population study by Tan et al. (40) whereby among the lean Singaporeans, Chinese were the most insulin sensitive and Indians were the least.

This present study has been one of the first attempts to investigate in details GLP-1 secretory capacity of various ethnicities across different glucose tolerance states. Our study revealed that ethnicity was a significant determinant of GLP-1 responses to OGTT, with Indians exhibiting significant positive correlation with GLP-1 levels. This is particularly evident in NGT and pre-DM groups. The augmented GLP-1 responses in Indians are significantly related to their high IR. High degree of IR triggers an increase in GLP-1 response as a compensatory mechanism to produce hyperinsulinemia (39). A similar finding by Slederring et al. (5) demonstrated that South Asians were more insulin resistant and had higher insulin levels but comparable glucose levels during an OGTT than Caucasians. This was associated with higher GLP-1 responses to OGTT in healthy South Asians compared with Caucasians. Furthermore, Tan et al. (40) found in parallel with high insulin resistance that Asian Indians had considerably higher β-cell function than Chinese and Malays, implying that compensatory hyperinsulinemia happens even in NGT individuals to maintain the fasting normoglycemia.

Our study also shows that Malays had the lowest GLP-1 secretion compared with Chinese and Indians. To date, there are limited incretin studies on the Malay population. Yeow et al. (36) recently examined GLP-1 responses and incretin effect of young onset T2DM in Malaysia who majority were Malays and reported that T2DM in the youth had similar GLP-1 concentrations to OGTT compared with control subjects with NGT. In comparison with Chinese, Malays exhibited less robust compensatory GLP-1 response. Our finding seems to corroborate the findings from Tan et al. (40) who reported that Malays had higher postprandial glucose excursion with lower disposition index that reflected inadequate β-cell secretory response relative to insulin sensitivity, compared with Chinese. It is worth mentioning that inter-ethnic disparities in the compensatory increase in GLP-1 secretion were not noticeable in the T2DM group.

### 4.4 Predictors of GLP-1 secretion

Multiple linear regression analysis demonstrated that age, ethnicity and systolic blood pressure were significantly correlated to the GLP-1 responses. Age was identified as a positive predictor of GLP-1 concentrations, and this was consistent with findings from other previous studies (18, 35, 41). This could be due to age-related decline in renal clearance of GLP-1 (24) or slower degradation of GLP-1 by DPP-IV (42).

Elevated systolic blood pressure is a risk factor of insulin resistance, which is the precursor to the development of pre-DM and T2DM. Both Wang et al. (20) and Zhang et al. (21) have demonstrated that increased blood pressure correlated with impaired GLP-1 secretion. In contrast to the earlier findings (21, 42), our results revealed that elevated systolic blood pressure was correlated with increased GLP-1 secretion. GLP-1 receptors are located ubiquitously in endothelial cells and vascular smooth muscles of the cardiovascular and renal system. Our finding is in agreement with Krisai et al. (43, 44) who demonstrated that GLP-1 level was strongly and positively correlated with both systolic and diastolic blood pressures. Several possible explanations include compensatory increase in GLP-1 level as an adaptive response to blood pressure elevation through the vasodilatory property of GLP-1, activation of renin–angiotensin system, and the microvascular endothelial dysfunction may cause insulin resistance, elevated blood pressure, and increased GLP-1 level (45).

In a number of Western studies, a higher BMI was associated with reduced GLP-1 response (18). However, consistent with other Asian studies, we did not find a correlation between BMI and GLP-1 secretion (7, 9, 46, 47). The BMI range between the NGT, pre-DM, and T2DM groups in our study showed considerable overlap, and this could explain the absence of any correlation between BMI and GLP-1 levels.

### 4.5 Study limitations and strengths

We omitted two doses of OGLDs; the two commonly used are metformin and gliclazide. The half-lives of metformin and gliclazide are 4–8 h and 10 h, respectively which translate into the five half-lives of 40 and 50 h, respectively. The metformin XR and gliclazide MR were administered daily. By omitting two daily doses (48 h), we would have covered the stipulated five half-lives for the drug to be cleared from the system so as not to influence the GLP-1 secretion. To minimize any possible confounding factors, we had made it compulsory that all patients were kept on a constant dose of the same OGLDs for the preceding 3 months prior to recruitment into the study and throughout the study period. Although there is a possibility of metformin increasing GLP-1 levels, a study by Vollmer et al. (35) have demonstrated that withdrawing metformin 2 days before the experiments showed no significant difference in the GLP-1 levels in patients treated with and without metformin. We had also performed a *post-hoc* analysis on the GLP-1 levels at 0, 30, and 120 min, ΔGLP-1_30 min_, ΔGLP1_120 min_ and AUC_GLP-1_ in metformin-treated or metformin-naive T2DM patients and noted no significant differences (data not shown).

The strength of the study lies in the fact that we were able to achieve the targeted sample size in the three glucose tolerance states with equal representation from each ethnic groups. In addition, we were able to recruit a diabetic population that has shorter duration of diabetes, well-controlled glycemia, and absence of any diabetic complications, hence minimizing any possible confounding factors. Increasing HbA1c level was found to be negatively associated with plasma GLP-1 responses (28). Therefore, we could not extend our findings to studies that included poorly controlled T2DM patients with relatively high HbA1c levels.

We also measured total GLP-1 and not the active, intact GLP-1 (7–36 amide) levels. The measurement of intact GLP-1 is compromised by its concentration below the detection limit of the assay due to its extremely rapid degradation by DPP-IV enzyme (48). Therefore, total GLP-1 (comprising both intact GLP-1 and its primary metabolite, GLP-1 9–36 amide) acts as a better indicator of the overall secretory response (48).

## 5 Conclusion

This is the first study that showed GLP-1 responses are augmented as IR states increase. Fasting and post-OGTT GLP-1 levels are raised in T2DM and pre-DM compared to NGT. These may represent an adaptive compensatory response that has not been described before. Among the three ethnic groups, the Indians have the highest IR state and the highest GLP-1 levels attesting to the adaptive compensatory nature of the GLP-1 secretion.

## Data availability statement

The original contributions presented in the study are included in the article/Supplementary Material. Further inquiries can be directed to the corresponding author.

## Ethics statement

This study was reviewed and approved by Research Ethics Committee of The National University of Malaysia (UKM PPI/111/8/JEP-2017-395); Medical Research and Ethics Committee, Ministry of Health Malaysia (NMRR-17-869-35075 (IIR)). The patients/participants provided their written informed consent to participate in this study.

## Author contributions

NK and NS conceived the idea and conceptualized the study. SC collected and analyzed the data. SC drafted the manuscript. NK, SR, KN, and NS reviewed the manuscript. All authors contributed to the article and approved the submitted version.

## Funding

This study was supported by a grant from The Universiti Kebangsaan Malaysia Medical Centre (GUP-2017-066) and a grant from the Malaysian Endocrine and Metabolic Society (L12-MEMS6).

## Conflict of interest

The authors declare that the research was conducted in the absence of any commercial or financial relationships that could be construed as a potential conflict of interest.

## Publisher’s note

All claims expressed in this article are solely those of the authors and do not necessarily represent those of their affiliated organizations, or those of the publisher, the editors and the reviewers. Any product that may be evaluated in this article, or claim that may be made by its manufacturer, is not guaranteed or endorsed by the publisher.
